# Cutaneous Melanomas Arising during Childhood: An Overview of the Main Entities

**DOI:** 10.3390/dermatopathology8030036

**Published:** 2021-08-01

**Authors:** Arnaud de la Fouchardière, Felix Boivin, Heather C. Etchevers, Nicolas Macagno

**Affiliations:** 1 Department of Biopathology, Center Léon Bérard, 69008 Lyon, France; felix.boivin@etu.univ-lyon1.fr (F.B.); nicolas.macagno@lyon.unicancer.fr (N.M.); 2 Cancer Research Center of Lyon, Equipe Labellisée Ligue Contre le Cancer, Université de Lyon, Université Claude Bernard Lyon 1, INSERM 1052, CNRS 5286, 69008 Lyon, France; 3 Marseille Medical Genetics, Institut MarMaRa, Aix-Marseille University, INSERM, U1251, 13005 Marseille, France; heather.etchevers@inserm.fr; 4 Department of Pathology, APHM, Timone University Hospital, 13005 Marseille, France

**Keywords:** congenital nevus, melanoma, childhood, skin, oncogenetics, SSM, malignant Spitz tumor

## Abstract

Cutaneous melanomas are exceptional in children and represent a variety of clinical situations, each with a different prognosis. In congenital nevi, the risk of transformation is correlated with the size of the nevus. The most frequent type is lateral transformation, extremely rare before puberty, reminiscent of a superficial spreading melanoma (SSM) ex-nevus. Deep nodular transformation is much rarer, can occur before puberty, and must be distinguished from benign proliferative nodules. Superficial spreading melanoma can also arise within small nevi, which were not visible at birth, usually after puberty, and can reveal a cancer predisposition syndrome (*CDKN2A* or *CDK4* germline mutations). Prognosis is correlated with classical histoprognostic features (mainly Breslow thickness). Spitz tumors are frequent in adolescents and encompass benign (Spitz nevus), intermediate (atypical Spitz tumor), and malignant forms (malignant Spitz tumor). The whole spectrum is characterized by specific morphology with spindled and epithelioid cells, genetic features, and an overall favorable outcome even if a regional lymph node is involved. Nevoid melanomas are rare and difficult to diagnose clinically and histologically. They can arise in late adolescence. Their prognosis is currently not very well ascertained. A small group of melanomas remains unclassified after histological and molecular assessment.

## 1. Introduction

Melanoma is a form of cancer arising from melanocytes that occurs mainly but not exclusively in the skin. Melanocytes are cells located in the basal layer of the epidermis that produce the melanin pigment responsible for skin color. They derive from neural crest progenitors (melanoblasts) that have migrated toward the skin during embryonic life. Melanocytes are nonadjacent and extend dendrites between the keratinocytes, through which a transcellular transfer of melanosomes encapsulating melanin occurs. This phenomenon *in fine* enables adjacent keratinocytes to protect their nuclei (i.e., DNA) from ultraviolet-induced mutations.

Nevi are benign melanocytic tumors of the skin defined by an abnormal number of melanocytes present in the epidermis and/or in the dermis, often arranged in “nests” where many melanocyte bodies are grouped in clusters and self-pigment. A small number of nevi can progressively undergo malignant transformation, usually through a multistep process that combines genetic (somatic and germline mutations), environmental (ultraviolet light exposure), and immunologic factors, to ultimately become malignant melanomas [1,2,3,4]. Children with a high count of nevi and a type I-II Fitzpatrick phototype or with a giant congenital melanocytic nevus (CMN) are the most at risk of developing melanoma. The WHO classification of skin tumors (4th edition) separates melanocytic proliferations into nine separate classes according to clinical, morphological, and genetic criteria [5].

If nevi are quite common in children, melanomas remain exceptional. They cannot be grouped under a single entity as they represent a variety of clinical situations, each with different prognoses. We will present an illustrated review depicting the specificities of each of the various settings in which cutaneous melanoma can arise during childhood. Nailbed, uveal, and mucous melanomas, which can exceptionally occur in children, will not be discussed.

## 2. Superficial Spreading Melanoma (SSM)

**Figure 1:**
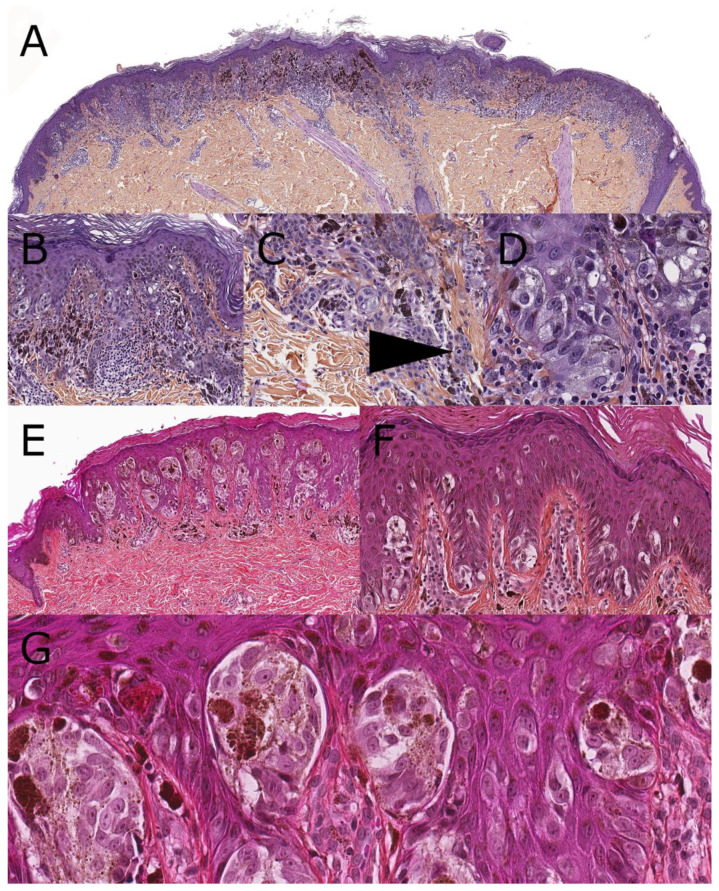
SSM melanoma and pagetoid Spitz nevus (hematoxylin, phloxin, safranin stain). (**A**): Low-magnification silhouette of SSM, 14-year-old: mainly junctional disorganized melanocytic proliferation. (**B**): Close-up view showing dispersed junctional nests. Ascent of isolated cell. Numerous lymphocytes and melanophages clutter the upper dermis. (**C**): Invasive melanoma with “pseudo-maturation” and mitotic figure (arrow). (**D**): Large, junctional, foamy melanocytes. (**E**): Low-magnification silhouette of a pagetoid Spitz nevus, 6-year-old. Epidermal hyperplasia encasing numerous small nests with a regular distribution. (**F**,**G**): Close-up view with isolated cells and small nests. An important pericellular retraction is seen. Ascent of small, isolated cells. Arborescent vascular structure in the upper dermis surrounded by small lymphocytes.

The occurrence of superficial spreading melanoma (SSM) is exceptional during childhood. Most often, the clinical setting is an adolescent with a type I-II phototype who presents with a progressive modification of a small nevus located on a sun-exposed area. A history of sunburns is common, on par with the phototype. The classic ABCDE rule applies only to this type of melanoma in children. These lesions are very similar to most of the SSMs found in adults. Histologically, a portion of the preexisting nevus can be visible if it has not been replaced by the invasive phase. Distribution is usually asymmetrical and/or haphazard with intraepidermal nests and isolated large melanocytes with a wide foamy/hyperpigmented cytoplasm. Lateral pagetoid scatter of such melanocytes is the histological hallmark of these tumors (Figure 1). In advanced SSM, the dermis is invaded, often by nests of large melanocytes with similar morphology. A dense lymphocytic infiltrate is commonly present on the invasive front of the tumor. These tumor-infiltrating lymphocytes (TILs) are in close connection with melanocytes (with cell-to-cell protein interaction). Breslow thickness, dermal mitotic activity, and epidermal ulceration are to be assessed. A *BRAF*.pV600E mutation is frequently present (>80%) both in the melanoma and the nevus from which it arose [4,6,7,8]. The need for an oncogenetic consultation must be evaluated according to the clinical setting. This will be discussed in the paragraph related to melanomas arising in the setting of a germline predisposition syndrome. The main differential diagnosis when no nevus is present is a pagetoid Spitz nevus (Figure 1).

## 3. Malignant Spitz Tumor (Melanoma with a Spitzoid Morphology and Specific Genetic Features)

Melanocytic neoplasms of the Spitz group show distinctive features and encompass benign, intermediate, and malignant tumors, all of which are characterized by prototypic enlarged spindle or epithelioid cells. Benign Spitz nevi are frequent during childhood, especially after puberty, and constitute a diagnostic pitfall of melanoma, mostly of the SSM-type. They often appear located on the lower limbs, especially the knee area, or on the face. Most are unique lesions, although rare cases of eruptive nevi have been described, notably in the clinical setting of a nevus spilus [9]. These lesions grow rapidly, and their important vascularization and lack of pigmentation clinically suggest a benign vascular tumor, such as a botryomycoma or an angioma. Spitz tumors encompass many morphological subtypes (pigmented, hyperpigmented spindle (Reed), plexiform, pagetoid, desmoplastic, angiomatoid…) and have specific, mutually exclusive genetic anomalies. These include *HRAS* mutations, receptor tyrosine kinase fusions, and serine-threonine kinase mutations and fusions, described in Table 1. For benign cases, the most common fusions involve *NTRK1/3*, *ALK1*, and *ROS1,* whereas MAP3K8 and ALK1 predominate in atypical and malignant Spitz tumors [6].

Histologically, Spitz nevi are symmetrical compound lesions with either a pediculated or elevated silhouette. The junctional component is arranged in nests that are often large, coalescent, and vertically oriented, intermingled with a hyperplastic epidermis, bearing pseudo-epitheliomatous changes. Depending on the histological subtype, cytology can range from small hyperpigmented spindled cells (Reed nevus prototypic morphology) to large epithelioid melanocytes with a glassy eosinophilic cytoplasm (Spitz nevus prototypic morphology), with every intermediate possibility. The dermal component is often of similar cytology with variable deep maturation according to the subtype. A fibrotic background with multiple dilated blood vessel lumens is commonly present in the upper dermis. Dermal mitotic activity is frequent (including atypical mitotic figures) on par with the rapid clinical growth.

**Figure 2:**
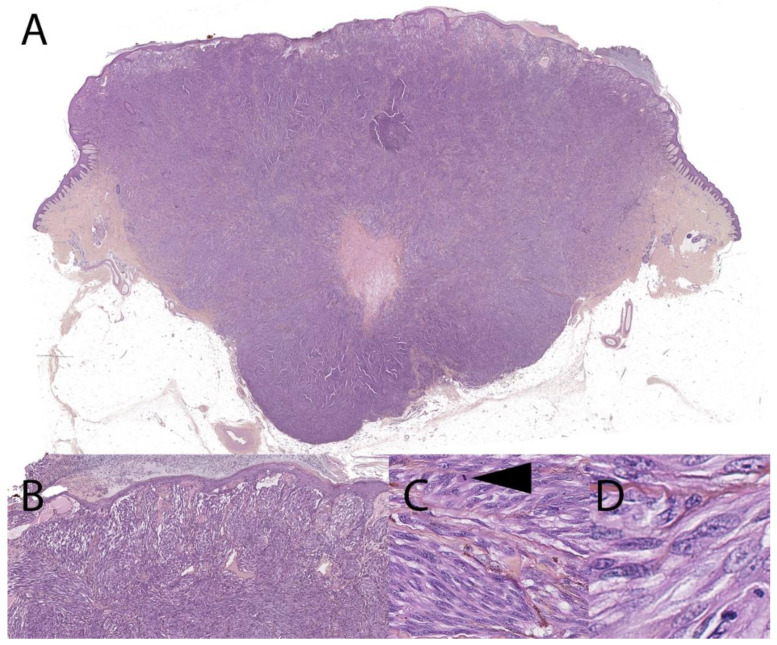
Malignant Spitz tumor, with *MYO5A-RET* fusion, 4-year-old (hematoxylin, phloxin, safranin stain). (**A**): Low-magnification silhouette: massive, sheet-like, destructive dermal expansion elevating the epidermis and invading the subcutis. Central dermal tumoral necrosis patch. (**B**): Close-up view of junction with a thinned epidermis covered by a crust. Obscuration of the grenz zone by dense fascicules. (**C**,**D**): High-power view of the dermal fascicules made of large spindled atypical melanocytes with mitotic activity (arrow).

In a small subset of cases and at all ages, a progression toward malignant melanoma can occur. Clinically, such lesions become rapidly bulky with heterochromatic melanocytic pigmentation. Histologically, a dermal clone appears, showing severe cytological atypia, increased mitotic activity, higher dermal density, a sheet-like pattern, and destructive extension to the subcutis (Figure 2). Importantly, Breslow thickness and nodal metastases do not carry the same weight in predicting outcome in pediatric Spitzoid neoplasms as in adult conventional melanoma. Indeed, regional nodal extension of a Spitz group neoplasm can commonly be observed without a dismal prognosis [33,34,35]. The diffuse widespread disease remains exceptional in cases with canonical genetic mutations. Homozygous deletions of *CDKN2A* and *TERT* promoter mutations are the most frequent secondary events observed during this progression [36].

The majority of pediatric melanomas arising from such lesions are associated with a good prognosis, even in cases with nodal extension [23]. Recent investigations recommend conservative management for pediatric Spitzoid tumors due to the extremely low associated death rate [33,37]. However, these studies were not always associated with a genetic analysis of the tumors. In the future, the identification of genetic Spitz-driving anomalies for each lesion could reveal itself useful to avoid overdiagnosis in pediatric populations while identifying the tumors the most at risk of a negative outcome. The recent advances in the knowledge of genetic alterations present in this group need to be explored in larger series. Indeed, there appears to be significant morphological overlap in lesions with similar genetic anomalies, including specificities related to the 5′ fusion partners, enhancing the complexity of this group. In our current vision, there could be significantly different outcomes in malignant Spitz tumors with tyrosine kinase fusion (*ALK, ROS1*, *NTRK1/3*) and serine-threonine fusion (*BRAF*, *MAP3K8*) with fatal cases published in the latter group only [23,36].

**Figure 3:**
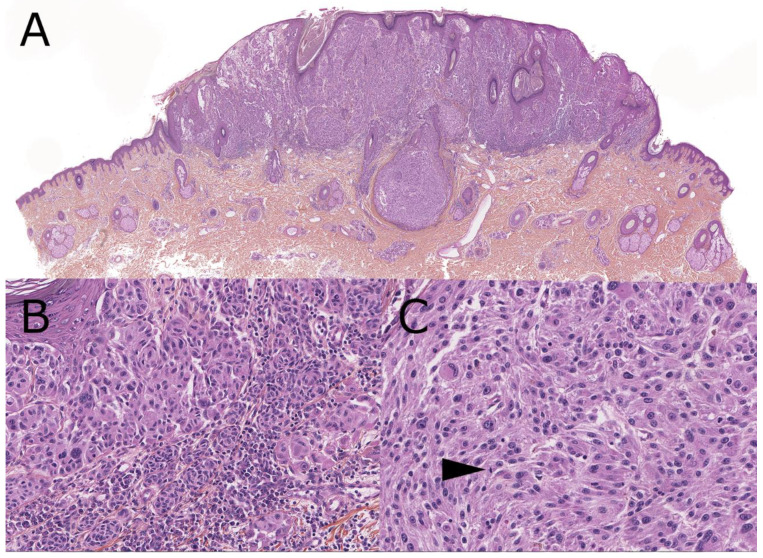
Spitzoid melanoma, 9-year-old (hematoxylin, phloxin, safranin stain). (**A**): Low-magnification silhouette: mainly dermal clonal proliferation, without pigmentation, elevating a slightly verrucous epidermis. Central vertical periadnexial expansion. (**B**): Confluent nests are present in the junction. Abrupt cytological hiatus and lymphocytes are seen in the dermis below. (**C**): Close-up view of the dermal component with spindled and epithelioid large melanocytes with mitotic figure (arrow).

As a side note, the “Spitzoid” terminology is used inconsistently and currently refers to cases with a Spitz-like cytomorphology, i.e., composed of large spindled or epithelioid melanocytes, but for which the specific genetic background linking it to the Spitz group has not been confirmed yet. Previous data have indicated that the Spitzoid histomorphology *per se* is an unreliable predictor of Spitz lineage, also encompassing *BRAF* or *NRAS*-mutated melanoma [38]. In other words, it means uncertainty about whether the prognosis will be good (*bona fide* Spitz, with tyrosine kinase fusion), or conversely, the evolution could be more aggressive and eventually lethal (*BRAF* or *NRAS* melanoma) (Figure 3). However, this morphological uncertainty has been dramatically reduced by various ancillary techniques aiming to reveal their genetic backgrounds, such as immunohistochemistry, FISH, and sequencing techniques (Table 1).

## 4. Melanoma Arising from a Congenital Nevus

Congenital melanocytic nevi (CMNi) are benign melanocytic tumors that can be present at birth or become apparent in early childhood. The size of these nevi ranges from a few millimeters to whole body segments. Genetic events occurring in melanoblasts or their immediate progenitors can explain the development of such lesions.

During embryogenesis, highly multipotent cells undergo a complex selective triage leading to the formation of a variety of cell lineages in the transient neural crest. Among these, melanoblasts later colonize the epidermis in which they become melanocytes, following earlier dorsal and later ventral migration schemes. Therefore, a single, multipotent neural crest stem cell bearing a somatic mutation can differentiate into a melanoblast-competent progenitor that, after population expansion and dissemination along peripheral nerves, will cause the formation of CMNiat the periphery. Due to the potentially early nature of this developmental anomaly, the resulting nevus may involve up to the whole thickness of the skin, but also the central nervous system (CNS). The risk of nevus deposits in leptomeninges increases when multiple lesions are observed in a newborn, a phenomenon sometimes called nevus satellitosis or neurocutaneous melanocytosis (NCM), but these are, in fact, simply disseminated nevi of identical genetic makeup to the largest CMN [39].

Activating missense mutations of *NRAS* (predominantly *NRAS* Q61K/L/R) are the most frequent genetic alterations associated with CMNi, found in 60% to 80% of the largest lesions [40,41]. Other anomalies such as the V600E mutation of *BRAF* and rarer gene rearrangements (*ZEB2-ALK*, *SOX5-RAF1*, *SAA6-RAF1*, and *BRAF*) have also been described [41,42,43].

Nonetheless, despite affecting the phenotype of the lesion, these alterations are not predictive of patient outcome [41]. Rather than genotype, the number of satellites (disseminated lesions) and the projected adult size (PAS) of the largest tumor are both linked to the risk of developing melanoma [44]. Therefore, small, solitary congenital nevi carry a lower risk of transformation than larger and multiple lesions. It is estimated that 1% of children worldwide are born with a visible nevus, but only 1/20,000 births will have a nevus over 10 cm, and as few as 1/500,000 births will have a nevus over 40 cm in PAS [45].

Both cutaneous and CNS sites can potentially give rise to melanomas in early childhood. The risk of transformation is most important in the first few years [46,47,48], but remains present throughout the whole patient’s life, with a lifetime risk of transformation estimated at up to 5% [49], thus requiring patient education about clinical and auto-evaluation methods on a regular basis.

Melanoma can develop from epidermal or dermal melanocytes of CMNi through lateral or deep nodular transformation, respectively.

**Figure 4:**
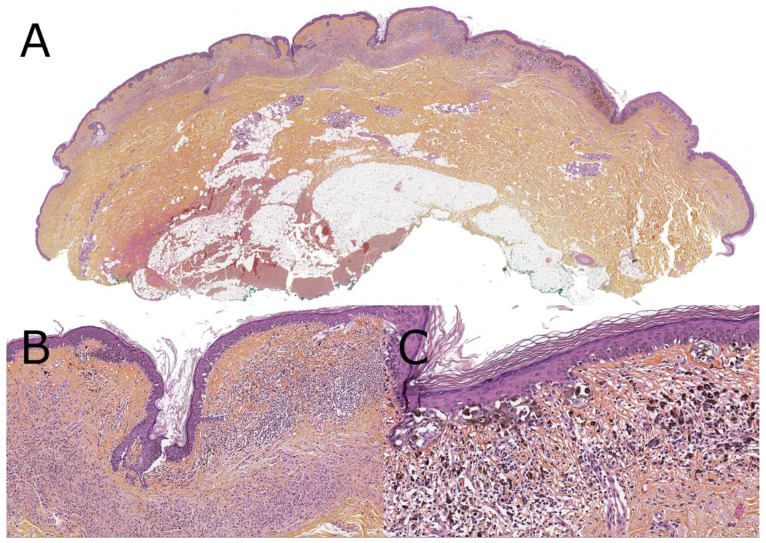
Superficial spreading melanoma ex-congenital nevus, 13-year-old (hematoxylin, phloxin, safranin stain). (**A**): Low-magnification silhouette: asymmetrical lesion with on the left side a compound, mainly dermal, congenital nevus with a loose horizontal band of melanocytes under a slightly verrucous epidermis, and on the right side a mainly junctional melanocytic proliferation underlined by a pigmented inflammatory reaction. (**B**): Close-up view on the transition between the nevus and melanoma. (**C**): Close-up view disorganized confluent junctional nests of large spindled hyperpigmented melanocytes in a slightly atrophic epidermis with numerous.

The most frequent is lateral transformation, which is fully reminiscent of a superficial spreading melanoma (SSM) *ex*-nevus (as described above). Such melanoma is virtually absent before puberty, except in patients with *xeroderma pigmentosum* (see below). Its low-magnification silhouette is typically asymmetrical: on one side of the nevus, an intraepidermal melanoma arises, first extending superficially, increasing the surface of the lesion in an asymmetrical and variably pigmented clinical pattern. Secondly, the proliferation will invade the dermis during the vertical phase. The prognosis of such melanoma parallels the prognosis of adult-type SSMs of identical Breslow thickness. Early recognition by regular skin screening is the most preventive method (Figure 4).

**Figure 5:**
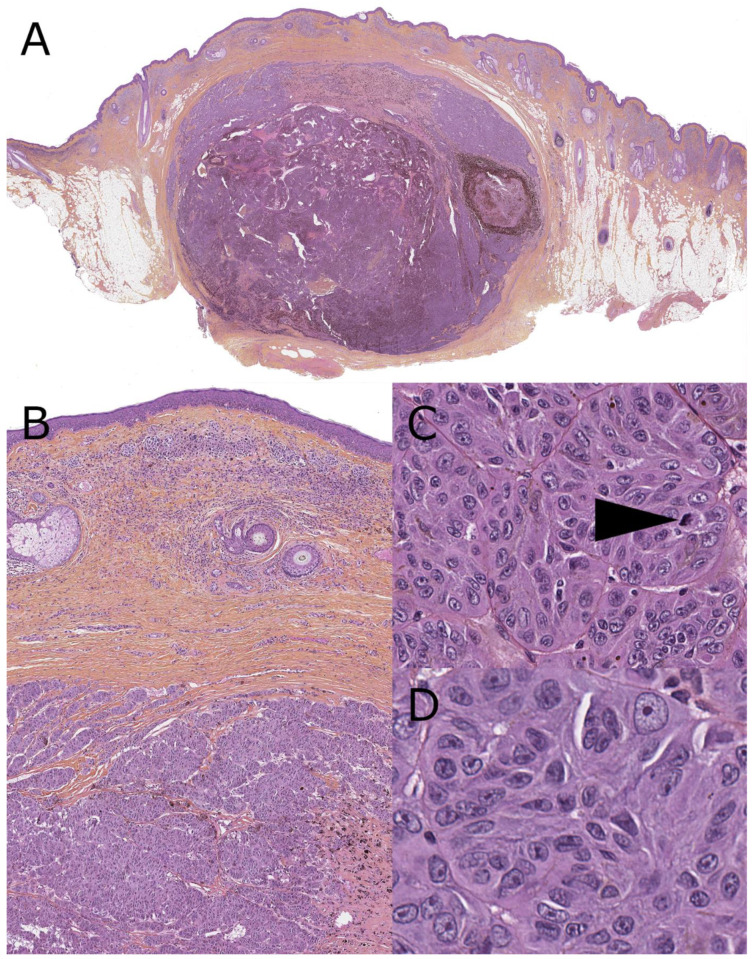
Melanoma ex-giant congenital nevus, 8-year-old (hematoxylin, phloxin, safranin stain). (**A**): Low-magnification silhouette: loose horizontal band of melanocytes under a verrucous epidermis elevated by a large cellular dermal nodule with hyperpigmented areas and patch of tumoral necrosis. (**B**): Close-up view on transition area with the congenital nevus made of loose bland melanocytes in the upper dermis separated by fibrous collagen from the dense nests of the melanoma underneath. (**C**,**D**): High-power view of the melanoma with confluent nests of large epithelioid and nevoid melanocytes; mitotic figure (arrow).

Deep nodular transformation can occur before puberty and is much rarer. Nodular transformation must be distinguished from benign proliferative nodules, which are more frequent. Indeed, rapidly growing, variably pigmented, and often multiple nodules can arise from giant CMNi during childhood before either stabilizing or becoming malignant. Nodular transformation usually shows higher mitotic activity and ulceration [48]. Moreover, melanoma and proliferative nodules seem to harbor different DNA-methylation patterns, suggesting a role for these studies in the differential diagnosis of such tumors [44]. In this context, epigenetic loss of H3K27me3 or 5-hmC expression, detected by immunohistochemistry, suggests a melanoma rather than a benign proliferative nodule [50,51,52]. Array-CGH can also be used to discriminate melanoma and proliferative nodules [53,54]. On prognostic grounds, the metastatic potential is greater in these melanomas, with a dismal prognosis and high mortality [50,55] (Figure 5).

Malignant transformation can also occur in the leptomeningeal CNS nevus deposits of NCM. They are usually detected at an advanced stage when neurological symptoms are triggered by tumor compression of adjacent structures and can be associated with hydrocephalus. At such stages of development, these melanomas are most often unresectable with a rapidly fatal outcome, and can disseminate through ventriculo-peritoneal shunts placed to treat the intracranial pressure [56,57,58]. However, CNS melanoma in a child with a large or giant CMN is not always fatal, and can develop in the presence of other brain malformations [59]. In evolved lesions, the presence of pigmented spindled and/or epithelioid cells in the cytology of cerebrospinal fluid is diagnostic [60].

## 5. Nevoid Melanomas

**Figure 6:**
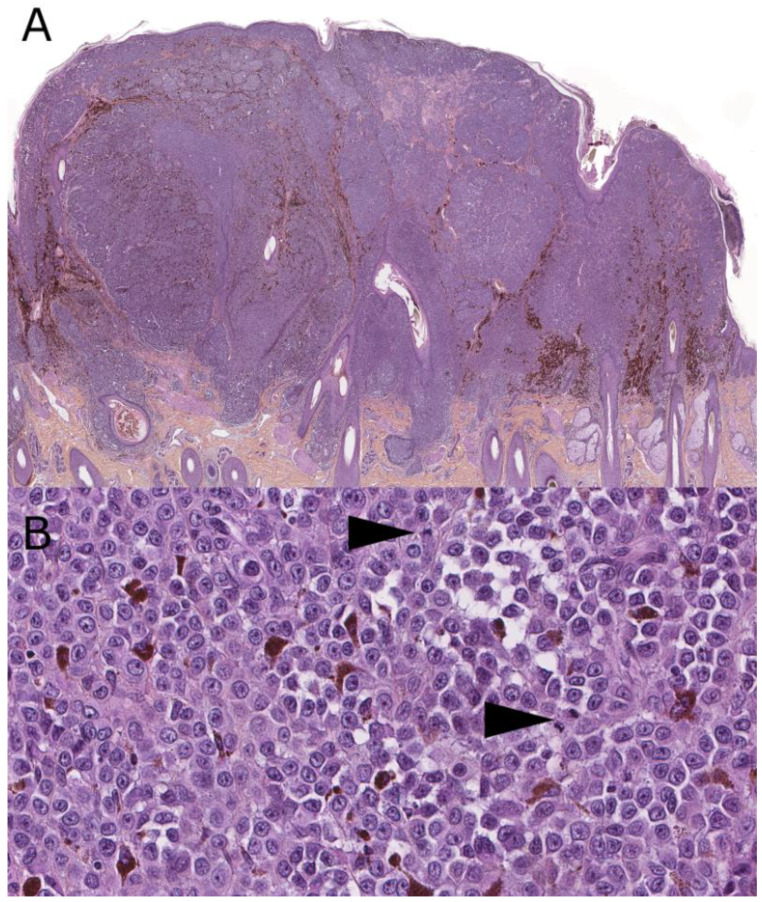
Nevoid melanoma, 17-year-old (hematoxylin, phloxin, safranin stain). (**A**): Low-magnification silhouette: massive dermal invasion by a pigmented clonal proliferation with the destruction of hair follicles. The grenz zone is obscured, and the epidermis is elevated. (**B**): Close-up view displaying sheets of large epithelioid melanocytes with mitotic figures (arrows) and dispersed melanophages.

Nevoid melanoma is rare and difficult to diagnose clinically as it usually grows slowly, simulating an exophytic, verrucous, warty nevus. Nevoid melanoma can, however, reach large clinical sizes. Histologically, nevoid melanoma constitutes an important diagnostic pitfall as it mimics a benign compound nevus, especially at low power. Indeed, nevoid melanoma provides the most important share of false-negative diagnoses of malignancy. A high density of melanocytes, an asymmetrical distribution of melanocytes, a perivascular and horizontal dermal extension that extends beyond the limit of the epidermal component are all suggestive of nevoid melanoma. Mitotic activity and Ki67 staining are frequently limited to the upper dermis. The immunophenotype is usually aberrant regarding the expression of Melan-A, HMB45, and p16. Nevoid melanoma mostly arises in adults, but it can also be observed in late adolescence (Figure 6). Their prognosis is currently not very well ascertained [61,62,63,64].

## 6. Melanomas Arising in the Setting of a Germline Predisposition Syndrome

A personal or familial history of previous melanoma is present in less than 10% of melanoma cases. Oncogenetic studies have identified several germline mutations that predispose to melanoma, even during childhood. Anomalies that only predispose adults to melanoma will, however, not be discussed.

Germline mutations in *CDKN2A* (encoding for the p16ink and p14arf proteins) are the most frequently (38%) found anomalies [65,66]. *CDKN2A* mutations are responsible for dysplastic nevus syndrome (OMIM #155601) in patients presenting a clinically large number of atypical nevi. In this setting, the risk of developing melanoma nears 100% during the person’s lifetime. This justifies recommending solar exposition avoidance as much as possible and skin surveillance starting at age 10. These melanomas occur mainly after puberty, as superficial spreading melanomas (SSM) arising from a preexisting compound nevus. This anomaly also predisposes to pancreatic adenocarcinoma, glioblastomas, sarcomas, and head and neck squamous cell carcinomas, but these arise in adults [67]. *CDK4* germline mutations are much less frequent and give rise to a similar clinical phenotype (multiple nevi with melanoma risk).

The terminology *xeroderma pigmentosum* encompasses a significant number of germline mutations impairing cellular DNA-repair functions to various degrees. In the most severe forms, children, who are exposed to sunlight, develop predominantly multiple basal cell and spindle cell skin carcinomas but also melanomas related both to chronic (*lentigo maligna* melanoma) and non-chronic sun exposure (SSM-type melanoma).

**Figure 7:**
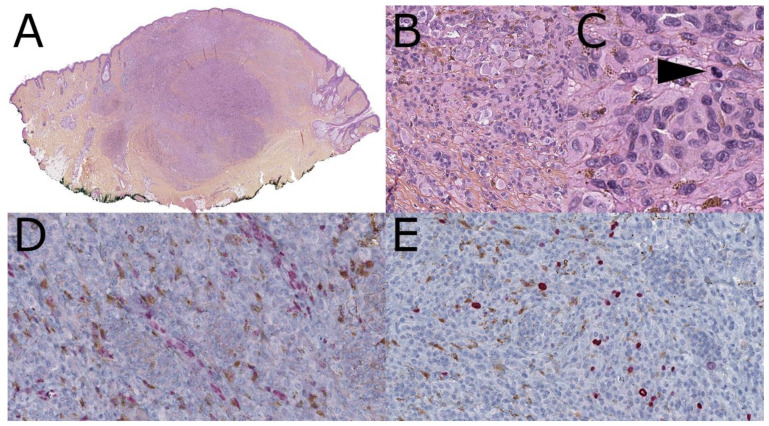
Melanoma *ex* BAP1 inactivated nevus; relapse, initial lesion at age 11(hematoxylin, phloxin, safranin stain). (**A**): Low-magnification silhouette: compound melanocytic proliferation with dense dermal nodular areas including pigmented clones. (**B**): Intermediate view of a cellular area with admixed small and large cells. (**C**): Close-up view of a deep dermal nodule with mixed small nevocytoid and large epithelioid melanocytes. A few of them show a pigmented cytoplasm; mitotic figure (arrow). (**D**): BAP1 immunohistochemistry (clone C4; 1/50): loss of nuclear staining with positive controls of endothelial cells. (**E**): Ki67 immunohistochemistry: variable dermal staining with clonal areas showing a 10% positivity.

The germline mutations of *BAP1* predispose to multiple cancers, including cutaneous and uveal melanomas. Most of these occur in adults. Carriers frequently develop benign BAP1-inactivated melanocytic nevi during childhood, which often helps identify the disease. Immunohistochemical studies can easily assess a loss of nuclear BAP1 expression. Exceptional transformations of such lesions can occur, including during childhood. The presence of a dense, possibly pigmented, clonal sheet-like area within a BAP1-inactivated nevus is evocative of this diagnosis (Figure 7).

Exceptional cases of melanomas and atypical melanocytic tumors have been described in children in the setting of a Li-Fraumeni syndrome (*TP53* mutation) [68,69].

## 7. Unclassified Melanomas

Despite the expansion of the histological and molecular classification of melanocytic tumors, some pediatric lesions do not fit in any of the categories due to their intradermal origin or as the consequence of undescribed clinical or genetic features. Such cases must be thoroughly explored on molecular grounds and clinically monitored. Research data are currently lacking in this field.

## 8. Melanoma Risk in Children with Impaired Immunity

It is now well known that immunosuppression, whatever the cause, is a risk factor for melanoma. Several studies have assessed the risk of developing melanoma in patients receiving immunomodulating drugs following organ or bone marrow transplantation. A case was recently reported where malignant leptomeningeal melanoma developed in an adolescent with CMNi, asymptomatic NCM and inflammatory bowel disease, following treatment with TNFα inhibitors [70]. Most such melanomas arise in adults, but skin and neurological surveillance is advised early on in this setting [71,72].

## 9. Conclusions

Numerous recent genetic discoveries and the WHO classification of skin tumors have restructured our vision of melanocytic tumors. Given the low frequency of pediatric melanoma, it may take some time for genetically well-characterized cohorts to be followed up and published. Nonetheless, this should encourage all teams involved in such rare cases to collaborate and work further to assess the presence of specific genetic markers that will ensure later more precise diagnosis, treatment, and follow-up.

## Figures and Tables

**Table 1:** Summary of the main genes involved in Spitz tumors, molecular alteration, and their function.

Gene	Molecular Alteration	Function	Frequence	References
*NTRK1*	Gene fusion	Receptor tyrosine kinase	Common	[10,11,12]
*HRAS*	Mutation amplification	Serine/Threonine kinase	Common	[13]
*ROS1*	Gene fusion	Receptor tyrosine kinase	Common	[14,15,16,17]
*ALK*	Gene fusion	Receptor tyrosine kinase	Common	[10,12,18,19]
*RET*	Gene fusion	Receptor tyrosine kinase	Rare	[10]
*NTRK3*	Gene fusion	Receptor tyrosine kinase	Common	[20,21]
*NTRK2*	Gene fusion	Receptor tyrosine kinase	Rare	[22]
*MAP3K8*	Gene fusion	Serine/Threonine kinase	Common	[23,24,25,26]
*BRAF*	Gene fusion	Serine/Threonine kinase	Uncommon	[12,25,27,28,29]
*MET*	Gene fusion	Receptor tyrosine kinase	Rare	[30,31]
*ERBB4*	Gene fusion	Receptor tyrosine kinase	Rare	[24]
*FGFR1*	Gene fusion	Receptor tyrosine kinase	Rare	[24]
*LCK*	Gene fusion	Tyrosine kinase	Rare	[9]
*MAP2K1*	Missense mutation	Serine/Threonine kinase	Rare	[32]
*MAP3K3*	Gene fusion	Serine/Threonine kinase	Rare	[24]
*MERTK*	Gene fusion	Receptor tyrosine kinase	Rare	[16]
*PRKDC*	Gene fusion	Serine/Threonine kinase	Rare	[24]
